# The Effect of Exercise-Based Interventions on Health-Related Quality of Life of Patients with Hematological Malignancies: A Systematic Review and Meta-Analysis

**DOI:** 10.3390/healthcare13050467

**Published:** 2025-02-21

**Authors:** Anita Borsati, Arianna Murri, Valentina Natalucci, Claudia Cerulli, Elena Barbieri, Francesco Lucertini, Massimo Lanza, Attilio Parisi, Christel Galvani, Pasqualina Buono, Annamaria Mancini, Francesco Fischetti, Luca Poli, Andrea Di Blasio, Alice Iannaccone, Alice Avancini, Caterina Mauri, Carlo Ferri Marini, Elisa Grazioli

**Affiliations:** 1 Biomedical, Clinical and Experimental Sciences, Department of Medicine, University of Verona, 37129 Verona, Italy; anita.borsati_02@univr.it; 2 Department of Neurosciences, Biomedicine and Movement, University of Verona, 37129 Verona, Italy; massimo.lanza@univr.it (M.L.); alice.avancini@univr.it (A.A.); 3 Department of Movement, Human and Health Science, University of Rome “Foro Italico”, 00135 Roma, Italy; arianna.murri@uniroma4.it (A.M.); claudia.cerulli@uniroma4.it (C.C.); attilio.parisi@uniroma4.it (A.P.); c.mauri@studenti.uniroma4.it (C.M.); 4 Department of Pathophysiology and Transplantation, University of Milan, 20133 Milan, Italy; valentina.natalucci@unimi.it; 5 Department of Biomolecular Sciences, University of Urbino Carlo Bo, 61029 Urbino, Italy; elena.barbieri@uniurb.it (E.B.); francesco.lucertini@uniurb.it (F.L.); 6 Exercise & Sport Science Laboratory, Department of Psychology, Università Cattolica del Sacro Cuore, 20162 Milan, Italy; christel.galvani@unicatt.it; 7 Department of Medical, Human Movement and Well-Being Sciences, University Parthenope, 80133 Napoli, Italy; pasqualina.buono@uniparthenope.it (P.B.); annamaria.mancini@uniparthenope.it (A.M.); 8 CEINGE-Biotecnologie Avanzate “Franco Salvatore”, 80131 Naples, Italy; 9 Department of Translational Biomedicine and Neuroscience (DiBraiN), University of Study of Bari, 70121 Bari, Italy; francesco.fischetti@uniba.it (F.F.); luca.poli@uniba.it (L.P.); 10 Department of Medicine and Aging Sciences, University “G. d’Annunzio” of Chieti-Pescara, 66013 Chieti, Italy; andiblasio@gmail.com; 11 Department of Human Sciences, Society and Health, University of Cassino and Southern Lazio, 03043 Cassino, Italy; alice.iannaccone@unicas.it; 12 Section of Innovation Biomedicine-Oncology Area, Department of Engineering for Innovation Medicine (DIMI), University of Verona and University and Hospital Trust (AOUI) of Verona, 37129 Verona, Italy; 13 Department of Human Movement Sciences, University of Groningen, University Medical Center Groningen, 9713 AV Groningen, The Netherlands; c.ferri.marini@umcg.nl

**Keywords:** exercise, quality of life, hematologic malignancies, complementary treatments, physical activity

## Abstract

**Background/Objectives**: Hematological cancers encompass a collection of heterogeneous conditions. The need for repeated treatments and prolonged hospitalization leads to a decrease in health-related quality of life. This systematic review and meta-analysis evaluate the effect of exercise interventions on functioning scales, global health status, and symptoms in patients with hematological cancers. **Methods**: PubMed, Web of Science, and Scopus databases were systematically screened, and randomized controlled trials were included. The primary outcome was health-related quality of life assessed through the European Organization for Research and Treatment of Cancer Quality of Life questionnaire. Meta-analysis was performed using a random-effects model and 95% confidence intervals. **Results**: A total of 14 trials involving 837 patients with mixed cancer types were included. Most studies evaluated exercise interventions during hospitalization, with aerobic and resistance training at moderate intensity being the most common modalities and adherence rates ranging from 28% to 100%. Exercise programs significantly improved physical (SMD 0.23; 95% CI: 0.06 to 0.40; *p* = 0.008), emotional (SMD 0.19; 95% CI: 0.03 to 0.36; *p* = 0.020) and cognitive functioning (SMD 0.20; 95% CI: 0.02 to 0.37; *p* = 0.026), and global health status (SMD 0.24; 95% CI: 0.03 to 0.46; *p* = 0.027). Significant reductions were observed in fatigue (SMD −0.33; 95% CI: −0.52 to −0.14; *p* = 0.001), pain (SMD −0.34; 95% CI: −0.53 to −0.15; *p* = 0.000), and insomnia (SMD −0.22; 95% CI: −0.41 to −0.03; *p* = 0.024). Heterogeneity was minimal across most domains, suggesting consistent findings. **Conclusions**: Exercise interventions are effective in enhancing functioning scales and global health status and reducing symptom burden in patients receiving intensive treatments. Future research is required to explore the long-term effects of exercise and develop tailored programs for specific hematologic malignancies and treatment settings.

## 1. Introduction

Hematological malignancies are defined as lymphatic and myeloid tumors caused by disruption of normal hematopoietic function [1]. Hematological cancers encompass a collection of heterogeneous conditions that can be classified into different subtypes, including leukemia, multiple myeloma, non-Hodgkin lymphoma, and Hodgkin lymphoma. Globally, the incidence of hematological malignancies has been increasing since 1990, reaching 134,385 patients in 2019; conversely, the age-standardized mortality rate for all types of these cancers has been declining [2]. This epidemiological picture is probably due to the development of new therapeutical options, as well as the optimization of existing therapies and supportive care aimed at both improving the quality of life and survival of patients [3]. Patients with hematological cancer frequently undergo repeated treatments and endure long hospital stays, often accompanied by periods of immobility. These circumstances can adversely affect their well-being, leading to fatigue, reduced energy levels, whole-body muscle weakness, heightened anxiety, and increased psychological stress. Ultimately, these factors contribute to a decline in health-related quality of life (HRQoL) [4]. HRQoL is defined as “a multidomain dynamic concept that represents the patient’s general perception of the effect of illness and treatment on physical, psychological, and social aspects of life” [5]. Quality of life is a multifaceted concept that encompasses various dimensions of human experience. In contrast, HRQoL focuses specifically on the impact of illness and its treatment on an individual’s well-being. HRQoL reflects the adaptive responses that a person has to the physical, mental, and social effects of disease, which in turn influences their overall satisfaction with life. Key dimensions of HRQoL include health perceptions, functional status, symptom burden, and individual preferences and values [6]. Given that HRQoL is recognized as a critical prognostic factor for survival, independent of clinical and socio-demographic characteristics known to influence disease progression, implementing multidisciplinary strategies to counteract cancer-related side effects and mitigate both physical and psychosocial deterioration is essential [7].

Physical activity and exercise represent promising interventions that can effectively support cancer treatment. These strategies are associated with enhancements in HRQoL, improvements in physical fitness, and reductions in symptoms such as fatigue, anxiety, and depression, both during and after the course of therapy [8,9]. In patients undergoing hematopoietic stem cell transplantation (HSCT), a supervised exercise program serves as a supportive treatment to address physical and psychosocial side effects. Evidence shows that regular exercise during and after transplantation can improve cardiorespiratory fitness, muscle strength, and functional mobility, resulting in better overall health outcomes [10,11]. Additionally, these interventions may help protect against complications related to the transplant and enhance recovery from side effects such as fatigue and decreased physical capacity. They also appear to positively impact bone marrow mobilization, hematopoietic recovery, and the overall success of the treatment [12]. One possible explanation is that physical activity and exercise may modulate the endogenous systemic environment, thereby influencing cellular processes and psychophysical health. The optimal exercise dose to achieve psychophysical benefits is not yet clear; however, the current exercise prescription guidelines for patients with cancer suggest at least 90 min of moderate-intensity aerobic activity per week, and resistance training at least two times a week for the main muscle groups (50–70% of one-repetition maximum−1 RM) [13]. However, these guidelines are built on evidence mostly derived by investigation on solid cancers and, in particular, on breast cancer settings, probably also due to the paucity of randomized controlled trials about exercise in patients with hematological malignancies.

Unfortunately, physical inactivity and sedentary are highly prevalent behaviors in patients with hematological cancers [14,15]. In addition, the potential impact of a structured physical exercise program, including type, intensity, duration, and the optimal time to start exercise intervention, on HRQoL in patients with hematological cancers remains unclear. Addressing this existing gap in literature, the present study aims to systematically summarize and quantify the impact of physical exercise on HRQoL in patients with hematological cancers, evaluated through the European Organization for Research and Treatment of Cancer Quality of Life questionnaire (EORTC QLQ C-30).

## 2. Materials and Methods

### 2.1. Objective and Outcomes

This systematic review and meta-analysis aimed to explore the effects of exercise on HRQoL in patients with hematological malignancies assessed with the EORTC QLQ C-30, a validated tool widely used in oncology research [16]. This questionnaire consists of 30 items covering five functional scales (physical, role, cognitive, emotional, and social), three symptom scales (fatigue, pain, nausea and vomiting), a global health and quality-of-life scale, and a financial impact assessment. Additionally, six single-item measures assess common cancer-related symptoms, including dyspnea, appetite loss, sleep disturbance, constipation, and diarrhea. Each item is rated on a 4-point Likert scale (1 = not at all to 4 = very much), with scores transformed to a 0–100 scale. Higher scores on the functional scales indicate better functioning, whereas higher scores on the symptom scales reflect a greater symptom burden. This meta-analysis specifically investigated the impact of exercise interventions on each scale of the EORTC QLQ C-30 compared to usual care. This systematic review was registered in the International Prospective Register of Systematic Reviews (PROSPERO) (ID: CRD42022347819), and the Preferred Reporting Items for Systematic Reviews and Meta-Analyses statement (PRISMA) were used to report the results [17].

### 2.2. Eligibility Criteria

Based on the PICO (Population, Intervention, Comparison, and Outcome) framework [18], study eligibility criteria were (i) having a randomized controlled trial design (RCT); (ii) including adult patients (age ≥ 18 years) with a diagnosis of hematologic malignancy; (iii) assessing the effect of an exercise intervention, defined as a planned, structured, and repetitive body movement to improve or maintain one or more components of physical fitness [19]; the type of physical exercise could be aerobic (i.e., any activity that uses large muscle groups, that can be maintained continuously, and is rhythmic in nature), resistance (i.e., a form of physical exercise that is designed to improve muscular fitness by exercising a muscle), or combined (i.e., aerobic plus resistance); (iv) compared to a non-exercise group (i.e., usual care) or general advice for physical activity, defined as any bodily movement produced by skeletal muscles that results in energy expenditure, or placebo interventions (i.e., stretching or relaxation); and (v) assessing the HRQoL through the EORTC QLQ C-30 questionnaire. Articles were excluded if (i) they had a non-English full text; (ii) the exercise intervention consisted of general physical activity recommendations/advice or physiotherapy involving passive techniques or specific exercises; or (iii) had a non-randomized controlled design (i.e., single-arm study, observational or cohort studies, quasi-experimental studies, qualitative studies, reviews, commentaries, and editorials).

### 2.3. Search Strategy

Systematic research was conducted from electronic databases PubMed/MEDLINE (National Library of Medicine), Scopus, and Web of Science in August 2022 and updated in May 2024. Three keywords have driven the research as follow: Physical Exercise (“Physical Activity” OR “Muscle Contraction” OR “Exercise” OR “Physical Exercise” OR “Exercise Therapy” OR “Resistance Training” OR “Walking” OR “Circuit-Based Training” OR “Strength Training” OR “Weight-Bearing Exercise” OR “Aerobic Training” OR “Cardiorespiratory Training” OR “Endurance Training” OR “Exercise Prescription” OR “Muscle Strength” OR “Proprioception” OR “Stretching” OR “Flexibility” OR “Yoga” OR “Tai Chi” OR ”Sport” OR “Dance” OR “Dancing” OR “Nordic Walking”); Cancer (“Lymphoma” OR “Leukemia” OR “Multiple Myeloma” OR “Hematological Malignancies” OR “Myeloma” OR “Hematologic Neoplasm” OR “Hematologic Cancer” OR “Hematologic Tumor”); and Quality of Life (“Quality Of Life” OR “Quality-Of-Life” OR “QOL” OR “Recurrence” OR “Relapse” OR “Side Effect” OR “Side-Effect” OR “Adverse Events” OR “Fitness” OR “Fatigue” OR “Adherence” OR “Sleep” OR “Body Composition”). Based on the eligibility criteria, the screening for titles/abstracts and subsequently for full-text were independently performed by A.B. and A.M., and a third author, E.G., resolved disagreements.

### 2.4. Data Extraction

Data were extracted from the articles included by two investigators (A.B. and A.M.) independently. The following data were extracted and summarized in an Excel spreadsheet (Microsoft, Redmond, WA, USA): information regarding the study (authors, design, country), participants (sample, size, gender, and age), medical factors (cancer type, treatment phase), exercise intervention (type, frequency, duration, intensity, length), type of intervention received by the control group (i.e., usual care, educational intervention or stretching), and mean and standard deviation or median and interquartile range for each EORTC QLQ C-30 domain of functioning and symptoms at baseline and after intervention.

### 2.5. Risk of Bias (Quality) Assessment

The quality of the RCTs was evaluated using the Cochrane risk of bias assessment tool (Cochrane Collaboration RoB 2.0) [20] covering five domains: (1) bias arising from the randomization process; (2) bias due to deviations from the intended interventions; (3) bias due to missing outcome data; (4) bias in measurement of the outcome; and (5) bias in selection of the reported result. Each domain was rated as having a “low risk of bias”, “some concerns”, or a “high risk of bias”. To determine the overall quality of each study, we followed the Cochrane guidelines: a study was classified as having a low risk of bias if all domains were judged as low risk, some concerns if at least one domain raised some concerns but no domain was at high risk, and high risk of bias if at least one domain was rated as high risk or if multiple domains raised concerns, potentially affecting the study’s validity. The study quality assessment for all included studies was independently and separately performed by two authors (A.B. and A.M.), and disagreements were resolved by a third author (E.G.).

### 2.6. Statistical Analysis

A qualitative synthesis was used to present data from the systematic review, as suggested by the Guidance on the Conduct of Narrative Synthesis in Systematic Reviews [21]. Subsequently, a meta-analysis was performed on studies reporting available data. When insufficient data were presented in the article, we tried to contact the authors to provide them. Changes in mean and standard deviation, from baseline to postintervention, for both groups were utilized to measure the effect size (ES). Due to the small sample size, ES, the associated standard error (SE), and the 95% confidence interval (CI) for each study were calculated using Hedges’ g. The pooled, sample-weighted, average ES values were computed using a random effect model and expressed the standardized mean difference (SMD) [22]. SMD values were interpreted according to Hedges’ g: 0.0 to ≤0.5 (small effect), 0.51 to 0.79 (moderate effect), and ≥0.8 (large effect) [22]. Statistical significance was set at a *p*-value < 0.05. Heterogeneity was analyzed using Cochran’s Q test and quantified by the I^2^ statistic and interpreted as follows: I^2^ <  25% (low levels of heterogeneity), 25–75% (medium levels of heterogeneity), and >75% (high levels of heterogeneity). Publication bias was assessed via visual inspection of a funnel plot and Egger’s test. Potential outliers were explored through a dedicated analysis by the leave-one-out method, i.e., omitting one study at a time. All analyses were conducted by IBM-SPSS-ver.29 (IBM Corp., Armonk, NY, USA).

## 3. Results

**Figure 1:**
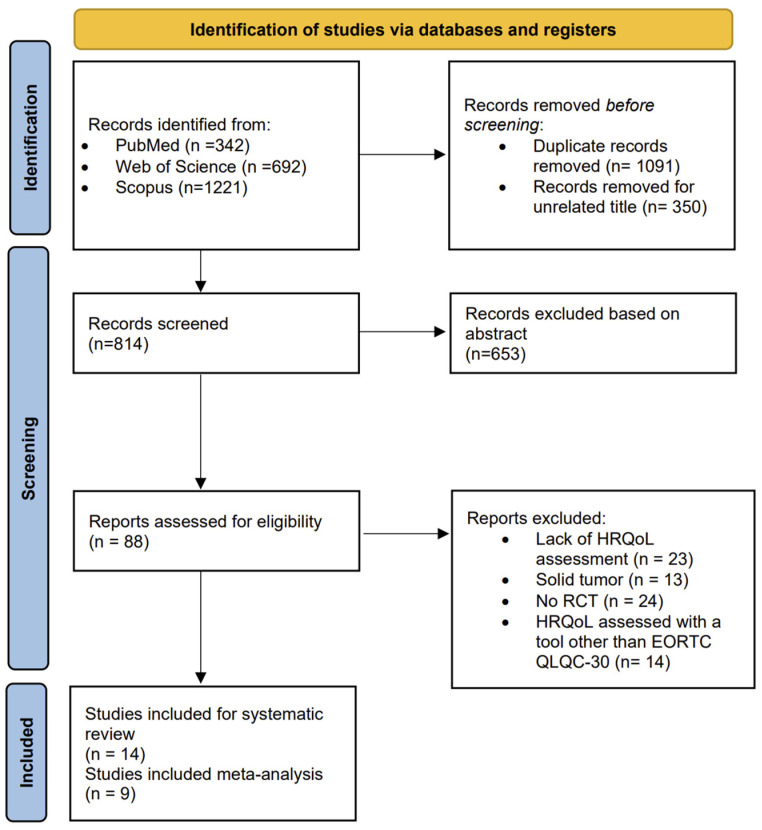
The PRISMA flow chart.

As reported in Figure 1, which displays the flow diagram of the screening process, a total of 2255 studies were identified from the literature search, and after removing duplicates, 1091 were selected. A total of 1003 articles were excluded after the title and abstract screening. The full texts of 88 potentially eligible studies were read, resulting in the exclusion of 74 studies at the full-text stage due to lack of HRQoL assessment, the use of a tool other than EORTC QLQ C-30 for HRQoL evaluation, the inclusion of patients with solid cancers, or a non-randomized controlled study design. Finally, 14 studies were included in the systematic review [23,24,25,26,27,28,29,30,31,32,33,34,35,36], and 9 had sufficient data to perform a meta-analysis [25,26,27,28,30,31,33,34,35].

### 3.1. Participants and Study Characteristics

Table 1 summarizes the characteristics of the included studies and patients. All the studies were two-arm RCTs [23,25,26,27,28,29,30,31,32,33,34,35,36], except for one study that exploited a three-arm design [24]. The overall sample consisted of 837 participants, 447 in the exercise group and 390 in the controls. The mean age of participants was 49.5 ± 6.7 years for the exercise group and 49.7 ± 6.4 years for the control group. Three out of fourteen trials exclusively targeted patients affected by acute myeloid leukemia [24,25,26] and two by different types of lymphomas [29,34]. Among the fourteen RCTs included, twelve explored the impact of an exercise program conducted during patients’ hospitalization for treatments, mostly chemotherapy and HSCT [23,24,25,27,28,29,30,32,33,34,35,36], while two studies were conducted on patients who had completed treatments [26,31].

### 3.2. Exercise Intervention Characteristics

The characteristics of the exercise programs are presented in Table 2. Seven studies were conducted in a hospital setting [24,25,27,28,29,30,32], three in gym-based settings [31,33,34], and four presented mixed characteristics, i.e., home-based plus supervised sessions [23,26,35,36]. Eleven investigations proposed a combined aerobic and resistance program [23,25,26,29,30,31,32,33,34,35,36], two, a combination of aerobic training and activities of daily living [27,28], and one study, aerobic and resistance training alone versus a control group [24]. The exercise frequency varied from 3–5 days for aerobic training and 2–3 times/week for resistance training. Similarly, the duration of the aerobic component ranged from 10 to 45 min for aerobic activities in ten studies, while only two studies reported the duration of resistance training [23,25].

For aerobic training, the intensity was monitored in 12 studies by different methods: the rate of perceived exertion (RPE) scale [25,35], the percentage of Watt max [27,28], based on maximal heart rate or heart rate reserve [23,24,29,30,31,34,36] and the percentage of maximal short exercise capacity [33]. A large majority of studies proposed moderate-intensity continuous aerobic training [23,24,25,27,28,30,31,34,35,36], one investigation a moderate-intensity interval training [29], and one a high-intensity interval training [29]. The intensity of resistance training was selected according to the RPE scale in four studies [24,30,35,36], testing the 1RM in three investigations [29,32,33,34], and not reported in three studies [23,25,31]. All the investigations proposed moderate-intensity resistance training.

The length of exercise intervention was less than 7 weeks in five studies [24,25,27,28,30,32] and more than 12 weeks in the others [23,26,29,31,33,34,35,36]. Adherence to the exercise programs ranged from 28% to 100%, and missed sessions were mainly due to medical contraindications, such as treatment-related side effects. The median dropout rate was 23% (IQR: 11–27.5%), and death, disease progression, and treatment-related complications were the most related reasons. Regarding safety, six studies reported no exercise-related adverse events [24,28,30,31,32,34], and two non-serious musculoskeletal side effects [25,33]. Two investigations described serious adverse events: one fall during the baseline six-minute walk test, resulting in a subarachnoid hemorrhage and two days of hospital surveillance [23], and two lumbar fractures [29]. More details of reasons for dropout and for missed exercise sessions, safety, and feasibility are presented in Tables S1 and S2 of Supplementary Materials.

### 3.3. Effects of Exercise on Functioning Scales and Global Health Status

Among the 14 studies included in the systematic review, three reported a significant improvement in physical functioning in the intervention arm compared to the control group [32,35,36]. In contrast, two studies observed a significant deterioration of physical functioning in both the exercise and control groups [27,28]. In the study of Cox et al., 2021, physical functioning improved in the exercise group but did not reach statistical significance, while it declined significantly in the control group [29]. Emotional functioning improved significantly in five studies after the exercise program [24,25,28,29,31]. Cognitive functioning increased in one study [25], although two studies showed a significant worsening of this outcome in the control group, with no significant changes observed in the exercise arm [27,29]. Except for the study of Cox et al., 2021, no significant improvements were observed for role and social functioning after the exercise intervention, and in the study of Baumann et al., 2010, role functioning decreased in both arms [27]. Global health status was higher in the exercise group in three studies compared to the control group [21,23,32].

**Figure 2:**
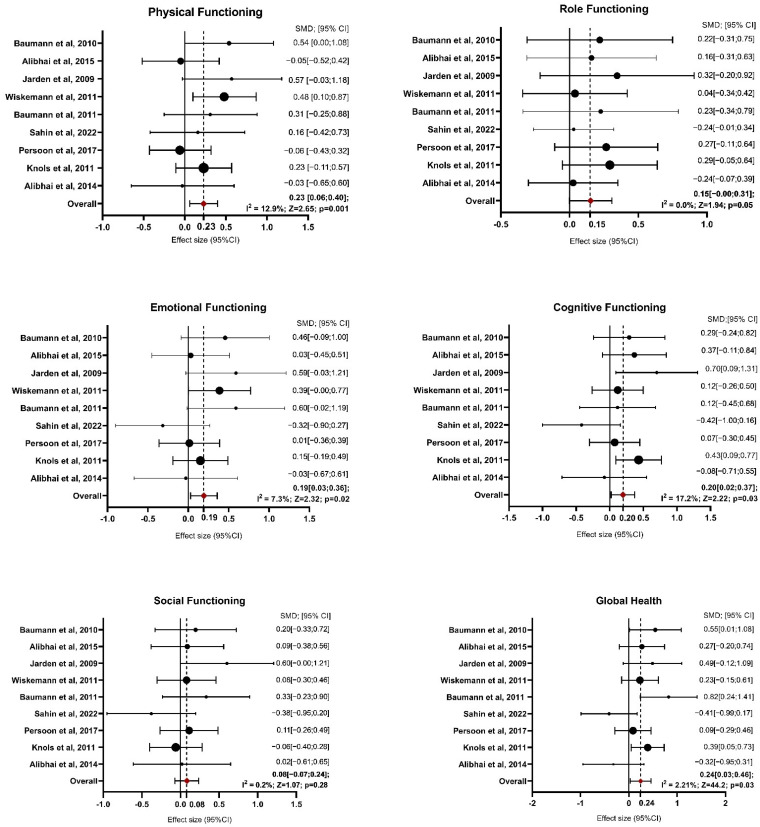
Forest Plot on Functioning Domains. Abbreviations—SMD: standardize mean difference; CI: confidence interval; Z: test for overall effect; I^2^: Heterogenity. Significative *p*-value ≤ 0.05. References: Baumann et al., 2010 [27]; Alibhai et al., 2015 [25]; Jarden et al., 2009 [30]; Wiskemann et al., 2011 [35]; Baumann et al., 2011 [28]; Sahin et al., 2022 [34]; Persoon et al., 2017 [33]; Knols et al., 2011 [31]; Alibhai et al., 2014 [26].

Concerning the quantitative synthesis, during the identification of outliers, conducted through graphical analysis of the funnel plot and analysis of heterogeneity measures, it emerged that the study by Santa Mina et al., 2020 was an outlier, possibly due to the smaller sample size. Specifically, the study of Santa Mina is an outlier for the physical functioning and role functioning domains, though not in other domains measured by the EORTC QLQ C-30 [23]. The ES related to the physical functioning domain is 0.03 with the study included, which is nearly negligible, whereas, without the study, it increases to 0.23, indicating a more substantial effect. Similarly, the I^2^ statistic drops from 87.2% (high heterogeneity) to 12.9% (low heterogeneity) when the study is removed, highlighting its significant contribution to the variability across studies. Therefore, it was decided to exclude it from the analysis of all domains. Table S3 of Supplementary Material reports detailed information on the results of functioning scales presented as tables, whereas Figure 2 shows the forest plot.

For physical functioning, the random-effects model estimated an average SMD of 0.23 (95% CI: 0.06 to 0.40), indicating that patients in the exercise group experienced greater physical functioning than controls after intervention (z = 2.654, *p* = 0.008). Heterogeneity was low, with an I^2^ of 12.9% (*p* = 0.379), suggesting that the findings were generally consistent across studies. For role functioning, the estimated average SMD was 0.15 (95% CI: −0.00 to 0.31), reflecting a marginal improvement due to exercise interventions, though the result was not statistically significant (*p* = 0.051). Heterogeneity was non-existent, with I^2^ at 0.0% (*p* = 0.751). The analysis of emotional functioning showed SMDs ranging from −0.32 to 0.60, with an average SMD of 0.19 (95% CI: 0.03 to 0.36), indicating a significant improvement in emotional functioning in response to exercise interventions (z = 2.328, *p* = 0.020). Heterogeneity was low (I^2^ = 7.3%, *p* = 0.257), implying consistency across studies. Regarding cognitive functioning, SMDs ranged from −0.42 to 0.43. The random-effects model estimated an average SMD of 0.20 (95% CI: 0.02 to 0.37), suggesting a significant improvement due to exercise (z = 2.222, *p* = 0.026) and heterogeneity was also low (I^2^ = 17.2%, *p* = 0.21). For social functioning, the SMDs ranged from −0.38 to 0.60. The average SMD was 0.08 (95% CI: −0.07 to 0.24), reflecting no significant effect of exercise interventions on social functioning (z = 1.074, *p* = 0.283). Heterogeneity was negligible, with I^2^ at 0.2% (*p* = 0.549). Global health improved significantly in the exercise group compared with the control group, with an estimated average SMD of 0.24 (95% CI: 0.03 to 0.46; *p* = 0.027). Moderate heterogeneity was observed (I^2^ = 44.2%, *p* = 0.058), suggesting some variability among the study results.

### 3.4. Effects of Exercise on Symptoms

Findings regarding symptoms varied across different exercise interventions. One study found a significant reduction in fatigue in the exercise group compared to a non-significant decrease in the control group [29]. Similarly, the study of Baumann et al., 2010 reported stable fatigue levels in the intervention arm, while the control group experienced a significant increase [27]. Regarding nausea and vomiting, two studies showed significant improvements compared to the usual care group [29,34]. Pain was significantly alleviated in two studies compared to the control group [29,35], although Baumann et al., 2010 found that pain significantly worsened in both arms [23]. Improvements in diarrhea were noted in two studies [30,31], with significant reductions reported in the exercise groups compared to controls. Only the study by Cox et al., 2021 demonstrated significant improvements in loss of appetite and constipation following the exercise intervention [29]. In contrast, no significant changes were observed in financial difficulties, dyspnea, or insomnia in any of the studies, either within the intervention arms or when compared to control groups.

**Figure 3:**
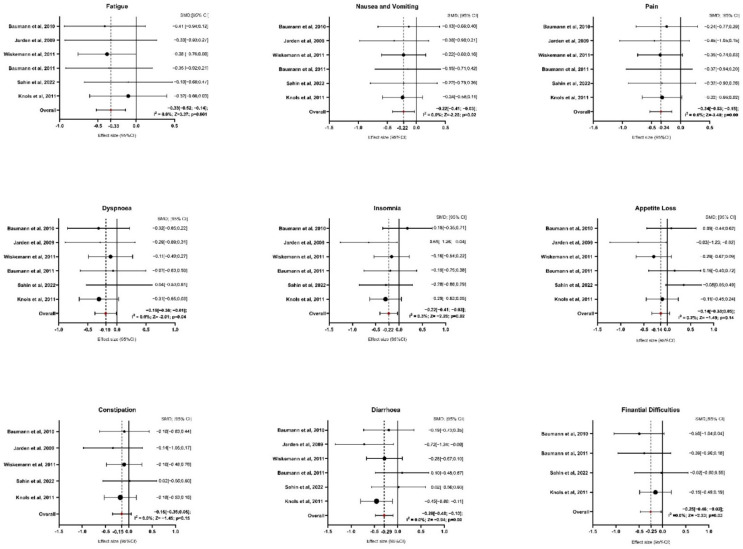
Forest Plot on Symptoms Domains. Abbreviations: SMD: standardize mean difference; CI: confidence interval; Z: test for overall effect; I^2^: Heterogenity. Significative *p*-value ≤ 0.05. References: Baumann et al., 2010 [27]; Jarden et al., 2009 [30]; Wiskemann et al., 2011 [35]; Baumann et al., 2011 [28]; Sahin et al., 2022 [34]; Knols et al., 2011 [31].

Six studies provided sufficient data for the analysis of symptom domains (Figure 3 and Table S4 of Supplementary Materials). Regarding fatigue, exercise interventions resulted in a significant reduction compared to the control group, with an average SMD of −0.33 (95% CI: −0.52 to −0.14; z = −3.370, *p* = 0.001). The analysis showed no heterogeneity (I^2^ = 0.0%, *p* = 0.979), indicating consistent results across studies. For nausea and vomiting, the average SMD was −0.22 (95% CI: −0.41 to −0.03), also showing a significant reduction with exercise interventions (z = −2.287, *p* = 0.022). Similar to fatigue, the results were homogenous (I^2^ = 0.0%, *p* = 0.993), suggesting that the outcomes were stable across the included studies. The analysis of pain showed a more pronounced effect, with an average SMD of −0.34 (95% CI: −0.53 to −0.15), highlighting a significant reduction in pain for participants in the exercise group (z = −3.485, *p* = 0.000). Again, there was no indication of heterogeneity (I^2^ = 0.0%, *p* = 0.997). In terms of dyspnea, the exercise interventions led to a significant reduction, with an average SMD of −0.19 (95% CI: −0.38 to −0.001; z = −2.016, *p* = 0.044). No heterogeneity was detected here either (I^2^ = 0.0%, *p* = 0.883), indicating similar results across studies. For insomnia, the average SMD was −0.22 (95% CI: −0.41 to −0.03), suggesting a significant improvement in sleep quality among participants who engaged in exercise (z = −2.264, *p* = 0.024). Heterogeneity was low, with I^2^ = 0.3% (*p* = 0.468), indicating consistent effects. The analysis of appetite loss showed an average SMD of −0.14 (95% CI: −0.33 to 0.05), indicating a non-significant result (z = −1.492, *p* = 0.136). Heterogeneity was minimal (I^2^ = 0.2%, *p* = 0.419), suggesting consistent findings across studies despite the lack of significance. Regarding constipation, after excluding the study of Baumann et al., 2011, five papers were included in the analysis [28]. The average SMD was −0.15 (95% CI: −0.35 to 0.05), showing no significant effect of exercise (z = −1.452, *p* = 0.147). The results were homogenous, with I^2^ = 0.0% (*p* = 0.858). In contrast, diarrhea showed a significant improvement with an average SMD of −0.29 (95% CI: −0.48 to 0.10; z = −2.941, *p* = 0.003). As with the other symptom domains, there was no heterogeneity (I^2^ = 0.0%, *p* = 0.349), indicating consistency among the studies. Finally, for financial difficulties, after excluding the study of Wiskemann et al., 2011 [35] and Jarden et al., 2024 [37], four studies were included in the analysis [27,28,31,34]. The average SMD was −0.25 (95% CI: −0.48 to −0.03), indicating a significant reduction in financial difficulties among participants who participated in exercise (z = −2.234, *p* = 0.025). There was no evidence of heterogeneity (I^2^ = 0.0%, *p* = 0.695), suggesting stable results across the studies.

### 3.5. Quality of Studies

The risk of bias assessment is summarized in Figure S1 of the Supplementary Material. Among the included studies, three demonstrated a low risk of bias, six had some concerns, and five were classified as having a high risk of bias, indicating a greater potential for compromised validity. The primary sources of bias were deviations from intended interventions (D2) and bias in the selection of the reported results (D5), which were frequently rated as high risk across multiple studies. These findings highlight the variability in study quality and underscore the need for cautious interpretation of the results.

## 4. Discussion

This meta-analysis sought to investigate the impact of exercise interventions on HRQoL in adult patients diagnosed with hematological malignancies. Our findings suggest that exercise programs have a beneficial impact on various HRQoL domains, including functioning and symptom management, particularly during the intensive treatment phase, such as chemotherapy or HSCT. This meta-analysis could offer valuable insights since HRQoL is recognized as a relevant topic in oncology, influencing treatment decisions and patient management strategies. Consequently, the development and implementation of interventions aimed at mitigating the adverse effects of anticancer therapies, while simultaneously enhancing HRQoL, are essential for optimizing patient outcomes. However, while robust evidence exists for the positive effects of exercise on HRQoL in patients with solid tumors, the impact on patients with hematological malignancies is still controversial. Our analysis indicated that exercise interventions significantly enhanced physical, emotional, and cognitive functioning, as well as overall health status among participants, yielding modest yet meaningful improvements. These findings align with a previous systematic review that identified increases in these outcomes in the experimental group compared to the control arm after exercise interventions, though these domains were assessed qualitatively [38]. Compared to another meta-analysis, our findings demonstrate a more pronounced effect on HRQoL. For instance, Yang et al., 2022 [39] examined the effects of exercise on HRQoL in seven RCTs, including 121 patients with mixed hematological cancers and thrombocytopenia. They observed significant enhancements only for emotional functioning (SMD = 12.34, 95% CI: 4.64 to 20.04, *p* < 0.01), GHS (SMD = 8.81, 95% CI: 1.81 to 15.81, *p* = 0.01) and pain (SMD = −12.77, 95% CI: −3.91 to −21.63, *p* = 0.005) for symptoms [39]. Similarly, another recent meta-analysis involving five studies reported moderate to small effects of exercise on global health status and physical functioning [37]. The reason for the results’ discrepancies in prior meta-analyses may be related to the limited number of studies included and the large heterogeneity of the samples. However, our results strengthen the evidence supporting the role of exercise in enhancing functioning and overall HRQoL during anticancer treatments. The observed enhancements in physical functioning, as evidenced in our review, are particularly noteworthy. These findings underscore the potential of exercise to mitigate the decline in physical abilities commonly experienced during treatment, particularly because of extended periods of immobilization associated with the inpatient phase. Moreover, the positive effects on emotional and cognitive domains suggest that exercise can help alleviate the psychological burden of cancer treatment, enhancing mood, emotional well-being, and cognitive function. Not surprisingly, the impact on role and social functioning was not statistically significant. This finding may reflect the challenges of maintaining social interactions during intensive treatment or hospitalization. In this sense, future research should explore how exercise programs can be tailored to promote social integration in patients who are hospitalized for treatments, perhaps by adding some group-based training, educational sessions, or involving family members in the exercise sessions.

Exercise interventions have been shown to significantly alleviate various cancer-related symptoms, such as fatigue, nausea, vomiting, pain, dyspnea, diarrhea, and insomnia. Our analysis, using the EORTC QLQ C-30 questionnaire, confirmed a significant reduction in fatigue following exercise compared to the control group. This is particularly important as fatigue, one of the most debilitating symptoms, severely impacts the HRQoL of patients, especially those undergoing HSCT, where fatigue can persist for years [40,41]. However, the effect of exercise on fatigue reduction in patients with hematological malignancies during treatment remains unclear. Our findings are in line with previous meta-analyses of Liang et al., 2018 [42] and Abo et al., 2021 [43], which demonstrated reduced fatigue levels in HSCT patients participating in exercise programs [42,43]. Abo et al., 2021 [43] found a significant improvement when using the EORTC QLQ C-30 and a not significant impact in studies that used different tools to assess fatigue [42]. In contrast, a recent meta-analysis revealed no significant changes in fatigue levels among patients with hematological malignancies after participating in aerobic or combined exercise interventions. It is important to note that this research aggregated data from 10 studies, utilizing a variety of questionnaires to evaluate fatigue, and included patients with different types of blood cancers who had not undergone HSCT [44]. The discrepancy in findings may be due to differences in the populations studied and the tools used to assess fatigue. Patients undergoing HSCT tend to be more responsive to exercise interventions because they typically start with worse fitness and higher fatigue levels. As a result, they are more likely to show significant improvements from baseline to endpoint, making changes in their fatigue more detectable in statistical analyses.

Our analysis revealed a consistent reduction in pain following an adapted exercise protocol, reinforcing the therapeutic role of exercise in managing cancer-related symptoms [13,45], as was evidenced in two meta-analyses. Jarden et al., 2024 [39] observed a significant moderate to small effect of exercise on pain levels in older patients undergoing antineoplastic treatments for hematological malignancies across 49 studies [39]. Similarly, Yang et al., 2022 [38] demonstrated pain reduction in the experimental group compared to controls, evaluated with the EORTC QLQ C-30, albeit the result was based on only three studies [38]. Regarding other domains, such as nausea, vomiting, diarrhea, dyspnea, and insomnia, fewer studies have specifically explored the effects of exercise on these outcomes in patients with hematological malignancies. One meta-analysis, which included 893 patients with mixed solid and blood cancer types, assessed the impact of exercise on cancer-related symptoms using the EORTC QLQ C-30. It found significant improvements in fatigue, pain, dyspnea, and insomnia but no significant effects on nausea, vomiting, loss of appetite, constipation, or diarrhea [46]. Thus, we hypothesize that exercise offers a promising non-pharmacological option for symptom management, potentially reducing the need for additional medications and enhancing treatment adherence and patient well-being. However, the severity of symptoms can vary greatly between patients and depend on the type of cancer therapy received. This variability likely contributes to the mixed results observed across different studies.

Our meta-analysis revealed a median adherence rate of 83% (IQR: 54.5–88%) and a completion rate of 81% (IQR: 73–88%) across the included studies. These results align with previous reviews, where adherence rates to exercise interventions ranged from 72% to 96% [39,42], with most RCTs concluding that their protocols were feasible. Additionally, only a few non-serious exercise-related adverse events were reported, a finding consistent with the review of Jarden et al., 2024 [39], which examined 49 studies involving 3494 older patients with hematological malignancies, and the review of Groβek et al., 2023 [47] assessing the feasibility of exercise in patients receiving chemotherapy [39,47].

However, although exercise interventions are claimed as feasible and safe in this setting, these conclusions should be approached with caution. Several important considerations must be addressed. First, the reported adherence rates often reflect the patients’ attendance at exercise sessions rather than the actual volume or intensity of the training performed. It is now well established that to improve health, it is not enough to follow a training protocol regularly but also to achieve an appropriate intensity and duration of training sessions. Unfortunately, many studies lack clarity on how intensity and adherence are monitored, raising concerns about the reliability of these data. Future research should prioritize rigorous monitoring to better understand the true impact of exercise on patients with hematological malignancies, especially in home-based or self-monitored programs outside the hospital setting. Another critical issue is the exclusion criteria used in these trials. As noted by Jarden et al., 2024 [39], many studies implemented stringent eligibility requirements, such as excluding patients with comorbidities, reduced functional capacity, or oncologist-determined medical contraindications [39]. While these precautions ensure patient safety, they also contribute to an underrepresentation of the “real-world” population of patients with hematological cancer, potentially biasing results. This could partly explain the suboptimal recruitment rates observed in several studies [23,26,31,33]. Unfortunately, the patients who might benefit the most from exercise interventions may be excluded from participation due to overly restrictive criteria. Lastly, while most studies claim their exercise protocols are safe, they often fail to provide detailed information about what happened during training sessions. Although adjustments to exercise intensity, duration, or type based on participants’ daily conditions (e.g., fatigue, fever, low platelet count) were commonly made, none of the studies offered a standardized or transparent summary of these adaptations. Such information is crucial not only for guiding future research but also for practitioners aiming to implement exercise safely in this population. Given the frequent safety-related concerns during treatment (e.g., anemia, infections), clear criteria and guidelines for making and reporting exercise adjustments are urgently needed. This would not only improve the quality of future studies but also provide a realistic depiction of how exercise interventions are managed in clinical practice.

A notable limitation of this analysis is the relatively small sample sizes in some studies, which may limit the generalizability of the findings. Additionally, most studies focused on exercise interventions during hospitalization, and only a few included cancer survivors. Furthermore, the limited number of studies available precluded subgroup analyses based on specific types of hematologic malignancies or treatment settings. Methodological limitations were also observed, with several studies exhibiting a high risk of bias, particularly concerning deviations from intended interventions and selective reporting of results. These factors may compromise the interpretability and validity of the findings.

Finally, significant heterogeneity across the included studies, arising from variations in exercise modalities and the methodological rigor of quality-of-life assessments, precluded definitive conclusions regarding the superiority of any specific training type. The search strategy, while utilizing three databases, may not have captured the entirety of relevant literature, potentially influencing the comprehensiveness of the review.

According to the current literature, exercise not only represents a promising approach for managing symptoms in adult patients with hematological cancer but also plays a crucial role in providing psychophysical benefits throughout both the disease-free continuum and the broader cancer continuum. However, barriers to initiating and maintaining an exercise regimen may vary depending on the stage of life. Findings from studies in adult patients with hematological malignancies suggest that exercise interventions should be structured and supervised to mitigate barriers contributing to increased sedentary behavior in this population. To go deeper, exercise protocols should be implemented immediately following diagnosis and throughout the disease course, both in hospital and outpatient settings.

Combined training (aerobic and strength exercises) is the most studied, while a more attractive and innovative approach seems to be essential, such as dance activities and/or digital activities, as has already been investigated in other cancer types [48,49]. Moreover, it could be useful for this population to find feasible methods to monitor hemoglobin status, facilitating better performance, as has already been analyzed in athletes [50].

## 5. Conclusions

Despite prevailing recommendations advising patients with hematological malignancies to rest and avoid intensive exercise due to treatment-related side effects, our findings indicate that exercise should be considered an essential component of supportive care during the treatment phase. The considerable variability in exercise parameters across studies—including intensity, duration, type, frequency, and supervision levels—poses a challenge in establishing an optimal exercise prescription for this population. However, evidence suggests that a combination of aerobic and resistance training, whether initiated during treatment, post-treatment, or continuously throughout the treatment trajectory, effectively mitigates physical deconditioning, alleviates symptom burden, and enhances health-related quality of life (HRQoL). Future research with larger sample sizes and rigorous methodologies is necessary to elucidate the long-term effects of exercise and to develop tailored intervention programs specific to distinct hematological malignancies and treatment settings.

## Figures and Tables

**Table 1:** Study design and participants’ characteristics.

Author, Year	Country	Sample Size (Allocation and Age)	Cancer Type	Anticancer Treatment Type	Timing of intervention *
Jarden et al., 2009 [30]	Denmark	42 pts EG: 21, age 40.9 ± 13.3 CG: 21, age 37.4 ± 11.1	Mixed hematological diseases	Allogenic HSCT	During
Baumann et al., 2010 [27]	Germany	64 pts EG: 32, age 44.9 ± 12.4 CG: 23, age 44.1 ± 14.2	Mixed hematological diseases	Chemotherapy/HSCT	During
Baumann et al., 2011 [28]	Germany	33 pts EG: 24, age 41.4 ± 11.8 CG: 23, age 42.8 ± 14.0	Mixed hematological diseases	Allogenic/autologous HSCT	During
Knols et al., 2011 [31]	Switzerland	131 pts EG: 64, age 46.7 ± 13.7 CG: 67, age 46.6 ± 12.0	Mixed hematological diseases	Chemotherapy/HSCT	After
Wiskemann et al., 2011 [35]	Germany	105 pts EG: 52, age 47.6 (18–70) CG: 53, age 50 (20–71)	Mixed hematological diseases	Allogenic HSCT	Before, during, and after
Alibhai et al., 2014 [26]	Canada	38 pts EG. 21, age 53.9 ± 8.2 CG: 17, age 58.8 ± 8.8	Acute myeloid leukemia	Chemotherapy/HSCT	After
Oechsle et al., 2014 [32]	Germany	48 pts EG: 24, age 51.7 ± 13.3 CG: 24, age 52.9 ± 15.4	Acute myeloid leukemia, multiple myeloma, non-Hodgkin’s lymphoma	Chemotherapy/HSCT	During
Alibhai et al., 2015 [25]	Canada	81 pts EG: 57, age 58 ± 13.9 CG: 24, age 52 ± 15.8	Acute myeloid leukemia	Chemotherapy	During
Persoon et al., 2017 [33]	Netherlands	109 pts EG. 54, age 53.5 (20–67) CG: 55, age 56 (19–67)	Multiple myeloma, non-Hodgkin’s lymphoma	Autologous HSCT	After
Wehrle et al., 2019 [24]	Germany	29 pts EG1: 8, age 47.7 (22–63) EG2: 6, age 47.4 (41–62) CG: 8, age 50.6 (35–58)	Acute myeloid leukemia	Chemotherapy	During
Santa Mina et al., 2020 [23]	Canada	30 pts EG: 15, age 50.4 ± 18.1 CG: 15, age 48.4 ± 13.0	Mixed hematological diseases	Allogenic HSCT	Before, during, and after
Yildiz Kabak et al., 2020 [36]	Turkey	50 pts EG: 25, age 51.6 ± 12.2 CG: 25, age 46.0 ± 16.6	Mixed hematological diseases	Allogenic/autologous HSCT	During and after
Cox et al., 2021 [29]	Italy	30 pts EG: 16, age 65.5 (34–77) CG: 14, age 63.0 (29–80)	Non-Hodgkin’s and Hodgkin’s lymphoma	Chemotherapy	During
Sahin et al., 2022 [34]	Turkey	47 pts EG: 28, age 41 ± 14 CG: 19, age 48 ± 20	Hodgkin’s lymphoma, non-Hodgkin’s lymphoma, small lymphocytic lymphoma and chronic lymphocytic leukemia	Chemotherapy/radiotherapy	During

RCT, randomized controlled trial; EG, exercise group; CG, control group; HSCT, hematopoietic stem cell transplant; pts, patients (* before, during, after anticancer treatment).

**Table 2:** Exercise intervention characteristics.

Study	Exercise Intervention						Control	Main Results
	*Type*	*Setting and* *Modality*	*Frequency*	*Intensity*	*DurATion of the Session*	*Length*	*Type of Intervention*	
Jarden et al., 2009 [30]	**AT:** cyclergometer **RT:** free hand and ankle weights, bicep curl, shoulder press, triceps extension, chest press, flyer, squat, hip flexion, knee extension, and leg curl and extension, core and back exercises. Dynamic and static stretching, psychoeducation, and progressive relaxation	Supervised at hospital	**AT:** 5/wk **RT:** 3/wk	**AT:** 50–75% HRmax **RT:** 1–2 sets 10–12 reps at 10–13 RPE	**AT:** 15–30 min **RT:** NR	4–6 weeks	Usual Care	*Within-group differences:* ↑ EG: diarrhea *Between-group differences*: No significant changes
Baumann et al., 2010 [27]	**AT:** cyclergometer **ADL:** walking, stepper, stretching, and coordination training	Supervised at hospital	5/wk, twice a day	**AT:** 80% of Watt max, increasing 25 W/2 min) **ADL:** stepping intervals 5 × 20 steps and 1 min rest at “slightly strenuous” to “strenuous” RPE	**AT:** 10–20 min **ADL:** 20–30 min	7 weeks (mean of 26 days)	Passive and active mobilization with a low intensity	*Within-group differences:* ↓ CG: GHS, PF, RF, and CF, fatigue, pain, insomnia, loss of appetite, diarrhea ↓ EG: PF, RF, loss of appetite, pain, diarrhea *Between-group differences:* ↑ EG vs. CG: GHS, PF
Baumann et al., 2011 [28]	**AT:** cyclergometer **ADL:** walking, stepper, stretching and coordination training	Supervised at hospital	5/wk, twice a day	**AT:** 80% of Watt max, increasing 25 W/2 min) **ADL:** stepping intervals 5 × 20 steps and 1 min rest at “slightly strenuous” to “strenuous” RPE	**AT:** 10–20 min **ADL:** 20–30 min	7 weeks (mean of 16 days)	Passive and active mobilization with a low intensity	*Within-group differences:* ↓ CG: PF, fatigue ↓ EG: PF ↑ EG: EF *Between-group differences:* No significant changes
Knols et al., 2011 [31]	**AT:** treadmill or cyclergometer **RT:** squats, step-ups, barbell rotations and upright rowing, chest press, triceps extension, biceps curl, modified curl-ups and calf raises with dumbbells or bodyweight	Supervised at gym	2/wk	**AT:** 50–80% HRmax **RT:** NR	**AT:** 10–30 min **RT:** NR	12 weeks	Usual Care	*Within-group differences:* ↑ EG: EF, diarrhea ↑ CG: EF *Between-group differences:* ↑ EG vs. CG: EF, diarrhea
Wiskemann et al., 2011 [35]	**AT:** treadmill, walking, or cyclergometer **RT:** elastic band exercises	Supervised at hospital + unsupervised at home	**AT:** 3–5/wk **RT:** 2/wk	**AT:** 12–14 RPE **RT:** 2–3 sets 8–20 reps at 14–16 RPE	**AT:** 20–40 min **RT:** NR	1–4 weeks before, during, and 6–8 weeks after HSCT	Physiotherapy, Physical Activity recommendations monitored by Pedometer, and weekly phone calls	*Within-group differences:* ↑ EG: PF, pain *Between-group differences:* ↑ EG vs. CG: PF, pain
Alibhai et al., 2014 [26]	**AT:** NR **RT:** exercises with bodyweight, stability ball, or elastic bands. Educational sessions	Supervised at hospital + unsupervised at home	3–5/wk at home 2/wk group sessions	**AT:** moderate intensity **RT:** moderate intensity	**AT:** 30 min **RT:** NR	12 weeks	Usual Care	*Within-group differences:* ↑ CG: GHS *Between-group differences:* No significant changes
Oechsle et al., 2014 [32]	**AT:** cyclergometer **RT:** bridging, sit-ups, arm and back exercises with elastic bands	Supervised at hospital	5/wk	**AT:** NR **RT:** 2 sets 16–25 reps at 40–60% 1RM	**AT:** 10–20 min **RT:** 20 min	4 weeks	Physiotherapy and Breathing exercises	*Within-group differences:* ↑ EG: PF *Between-group differences:* ↑ EG vs. CG: PF
Alibhai et al., 2015 [25]	**AT:** treadmill, walking, or cyclergometer **RT:** bodyweight, elastic band, or free weight exercises	Supervised at hospital	4–5/wk	**AT:** 3–6 RPE **RT:** NR	**AT:** 10–40 min **RT:** 10–25 min	Mean of 36 days	Usual Care	*Within-group differences:* ↑ EG: GHS, EF ↑ CG: GHS, SF *Between-group differences:* ↑ EG vs. CG: CF
Persoon et al., 2017 [33]	**AT:** cyclergometer **RT:** vertical row, leg press, bench/chest press and pull over/flies, abdominal muscles and the upper legs	Supervised at gym	2/wk (1–12 weeks) 1/wk (13–18 weeks)	**AT:** interval training 30–65% of maximal short exercise capacity **RT:** 2 sets 10 reps at 65–80% 1RM (1–12 weeks) and 2 sets 20 reps at 35–40% 1RM (13–18 weeks)	NR	18 weeks	Usual Care	*Within-group differences:* No significant changes *Between-group differences:* No significant changes
Wehrle et al., 2019 [24]	**AT:** treadmill or cyclergometer **RT:** bodyweight or dumbbell/elastic band exercises	Supervised at hospital	3/wk	**AT:** 60–70% HRmax **RT:** 12–14 RPE	**AT:** 30–45 min **RT:** NR	4 weeks	Stretching and Mobilization at low intensity	*Within-group differences:* ↑ AT: EF ↑ RT: EF *Between-group differences:* No significant changes
Santa Mina et al., 2020 [23]	**AT:** treadmill, walking, elliptical machine or cyclergometer **RT:** lower body (squats, side squats, and lunges), upper body (back rows, chest fly, chest press, push up, shoulder press, lateral arm raises, biceps curls, and triceps extensions), and core exercises with free weights, elastic bands, or bodyweight. Relaxation exercises	Supervised at hospital + unsupervised at home	3/wk	**AT:** 60–80% HRR (before and after), 60% HRR (during) **RT:** 8–10 exercises, 2–3 sets of 6–15 reps (before and after), 1–2 sets 4–6 reps (during)	**AT:** 10–15 min (before, during, and after) **RT:** 30–45 min (before and after), 10–30 min (during)	1–8 weeks before, during, and 100 days after HSCT	Usual Care	*Within-group differences:* No significant changes *Between-group differences:* ↑ EG vs. CG: QLQ-C30 summary score after 1 year
Yildiz Kabak et al., 2020 [36]	**AT:** walking or cyclergometer **RT:** upper and lower muscles body weight, manual resistance, or elastic band exercises. Relaxation exercises	Supervised at hospital + unsupervised at home	5/wk, once or twice a day	**AT:** 40–60% HRmax **RT:** 1–2 sets 10–15 reps at 10–13 RPE	**AT:** 25–30 min **RT:** NR	4 weeks during and 100 days after HSCT	Usual Care	*Within-group differences:* ↑ EG: general health score, functioning score, symptoms score *Between-group differences:* ↑ EG vs. CG: functioning score, symptoms score
Cox et al., 2021 [29]	**AT:** treadmill or cyclergometer **RT:** shoulder press, bench press, lat machine, abdominal crunch, leg press, leg extension. Balance training	Supervised at hospital	2/wk	**AT:** continuous 55–70% HRmax the 1st week, 5 intervals of 1 min at 80–90% HRmax from the 2nd **RT:** 3 exercises 1–3 sets 10 reps at 60–80% 1RM	**AT:** 10–20 min **RT:** 10–20 min	16 weeks	Motivational interview and exercise booklet	*Within-group differences:* ↑ EG: RF, EF, SF, fatigue, nausea and vomiting, pain, appetite loss and constipation ↓ CG: PF, RF, EF, CF, SF *Between-group differences:* ↑ EG vs. CG: PF, RF, EF, CF, SF and fatigue
Sahin et al., 2022 [34]	**AT:** treadmill **RT:** chest press, leg flexion, triceps press, leg extension, biceps exercise with dumbbell or instrument and leg press	Supervised at gym	3/wk	**AT:** running at 60% HRR **RT:** 3 rounds at 60% 1RM	**AT:** 15 min **RT:** 30 min	16 weeks	Exercise counseling, Exercise log	*Within-group differences:* ↑ EG: nausea and vomiting *Between-group differences:* No significant changes

Abbreviations and symbols: ↑ significant improvement; ↓ significant worsening. EG exercise group; CG control group; ADL activities of daily living; AT aerobic training; RT resistance training; HRR heart rate reserve; RM repetition maximum; NR not reported; EORTC QLQ C-30 European Organization for the Research and Treatment of Cancer Quality of Life Questionnaire; RPE rate of perceived exertion; PF physical functioning; EF emotional functioning; CF cognitive functioning; RF role functioning; SF social functioning.

## Supplementary Materials

The following supporting information can be downloaded at: https://www.mdpi.com/article/10.3390/healthcare13050467/s1, Table S1. Reasons for dropout and for missed exercise sessions; Table S2. Feasibility and safety of the studies included; Figure S1. Traffic light plot for risk of bias assessment; Table S3. Meta-analysis results on the effects of exercise on functioning domains and global health status of EORTC QLQ-C30; Table S4. Meta-analysis results on the effects of exercise on symptoms domains of EORTC QLQ-C30.
