# Supplementary material for: The Effect of Exercise-Based Interventions on Health-Related Quality of Life of Patients with Hematological Malignancies: A Systematic Review and Meta-Analysis
Source: Healthcare (Basel). 2025 Feb 21;13(5):467. doi: 10.3390/healthcare13050467 (PMC11898443; doi:10.3390/healthcare13050467)
Supplement: Supplementary file 1 [file healthcare-13-00467-s001.zip › healthcare-3446657-supplementary.pdf]

Supplementary Materials

Table S1. Reasons for dropout and for missed exercise sessions

Table S2. Feasibility and safety of the studies included

Figure S1. Traffic light plot for risk of bias assessment

Table S3. Meta-analysis results on the effects of exercise on functioning domains and global health status of EORTC QLQ-C30

Table S4. Meta-analysis results on the effects of exercise on symptoms domains of EORTC QLQ-C30

Table S1. Reasons for dropout and for missed exercise sessions

Author, year	Reasons for missed sessions	Reasons for dropout
Jarden et al, 2009 [30]	NR	Death or transplant-related complications
Baumann et al, 2010 [27]	• Medical reasons: acute bleeding, platelet count <10 mcL, haemoglobin levels <80 g/L, strong pain, altered consciousness, somnolence, confusion, poor blood circulation, dizziness, temperature >38 C, severe infection, nausea and vomiting	Death from transplant-related complications or disease progression
Baumann et al, 2011 [28]	• Medical reasons: acute bleeding, platelet count <10 mcL, haemoglobin levels <80 g/L, strong pain, altered consciousness, somnolence, confusion, poor blood circulation, dizziness, temperature >38 C, severe infection, nausea and vomiting	Death from transplant-related complications or disease progression
Knols et al, 2011 [31]	NR	Disease progression, lack of interest, comorbidities, not agree with allocation
Wiskemann et al, 2011 [35]	• Medical reasons: acute bleeding, platelet count <10 mcL, haemoglobin levels <80 g/L, strong pain, altered consciousness, somnolence, confusion, poor blood circulation, dizziness, temperature >38 C, severe infection, nausea and vomiting	Death or unknown
Alibhai et al, 2014 [26]	NR	Disease relapse or unknown
Oechsle et al, 2014 [32]	NR	Death for treatment-related complications, disease progression, change to allogeneic stem cell transplantation or palliative treatment or personal reasons
Alibhai et al, 2015 [25]	• Medical reasons: fatigue, pain, delay in transfusion, fever, weakness, nausea/vomiting, bleeding, diarrhea, sleeping, headache, transfusion-related symptoms, dizziness<br>Personal reasons	Death or unknown

Persoon et al, 2017 [33]	• Medical reasons: illness or injuries<br>• Personal reasons: holidays<br>• Session took place after post-intervention assessment	Disease progression, disease relapse, personal reasons
Wehrle et al, 2019 [24]	• Medical reasons: acute bleeding, platelet count <10 mcL, haemoglobin levels <80 g/L, strong pain, altered consciousness, somnolence, confusion, poor blood circulation, dizziness, temperature >38 C, severe infection, nausea and vomiting<br>• Personal reasons: lack of time	Mental overload, change in diagnosis, persistent thrombocytopenia, death
Santa Mina et al, 2020 [23]	NR	Death, disease relapse, non-eligible for transplant, missed appointment, not physically able, personal reasons
Yildiz Kabak et al, 2020 [36]	NR	NR
Cox et al, 2021 [29]	NR	Vertebral compression fractures, hip/back pain, inguinal hernia, chemotherapy adverse events, personal reasons
Sahin et al, 2022 [34]	• Medical reasons: fatigue, generalized somatic pain, febrile reactions after chemotherapy, overlap of chemotherapy and exercise schedules<br>Personal reasons: social and financial issues	NR
**Abbreviations:** NR, not reported		

Table S2. Feasibility and safety of the studies included

Author, year	Recruitment rate (%)	Completion rate (%)	Adherence (%)	Dropout (%)	Compliance (%)	EX-related adverse events (n, type)
Jarden et al, 2009 [30]	51	81	90	19	NR	0

Baumann et al, 2010 [27]	NR	77	NR	23	NR	NR
Baumann et al, 2011 [28]	NR	70	NR	30	NR	0
Knols et al, 2011 [31]	42	87	85	11	NR	0
Wiskemann et al, 2011 [35]	94	76	87	24	NR	NR
Alibhai et al, 2014 [26]	38	95	28	10	NR	NR
Oechsle et al, 2014 [32]	NR	83	NR	17	NR	0
Alibhai et al, 2015 [21]	56	86	54	25	NR	4, musculoskeletal events (non-serious)
Persoon et al, 2017 [25]	23	89	86	11	NR	1, strained calf muscles (non-serious)
Wehrle et al, 2019 [24]	74	76	AT: 69<br>RT: 76	24	NR	0
Santa Mina et al, 2020 [23]	20	33	Prehab: 56 (AT); 99 (RT)<br>Inpatient: 55 (AT); 99 (RT)	60	NR	1, subarachnoid haemorrhage and admission to the hospital (serious)

			Rehab: 20 (AT); 100 (RT)			
Yildiz Kabak et al, 2020 [36]	82	NR	89	NR	NR	NR
Cox et al, 2021 [29]	86	50	50	50	NR	2, lumbar compression fractures (serious)
Sahin et al, 2022 [34]	69	100	83	0	NR	0

Abbreviations: *AT*, aerobic training; *RT*, resistance training; *NR*, not reported

Definitions: *Recruitment rate*: ratio of patients enrolled compared with the number of eligible patients; *Completion rate:* ratio of patients who completed the exercise intervention out of the enrolled; *Adherence:* the number of exercise sessions attended out of the total planned sessions; *Compliance:* ratio of the total volume of exercise completed compared with the prescribed one; *EX-related adverse events:* number of adverse events related to exercise participation (sessions or assessments).

Figure S1. Traffic light plot for risk of bias assessment

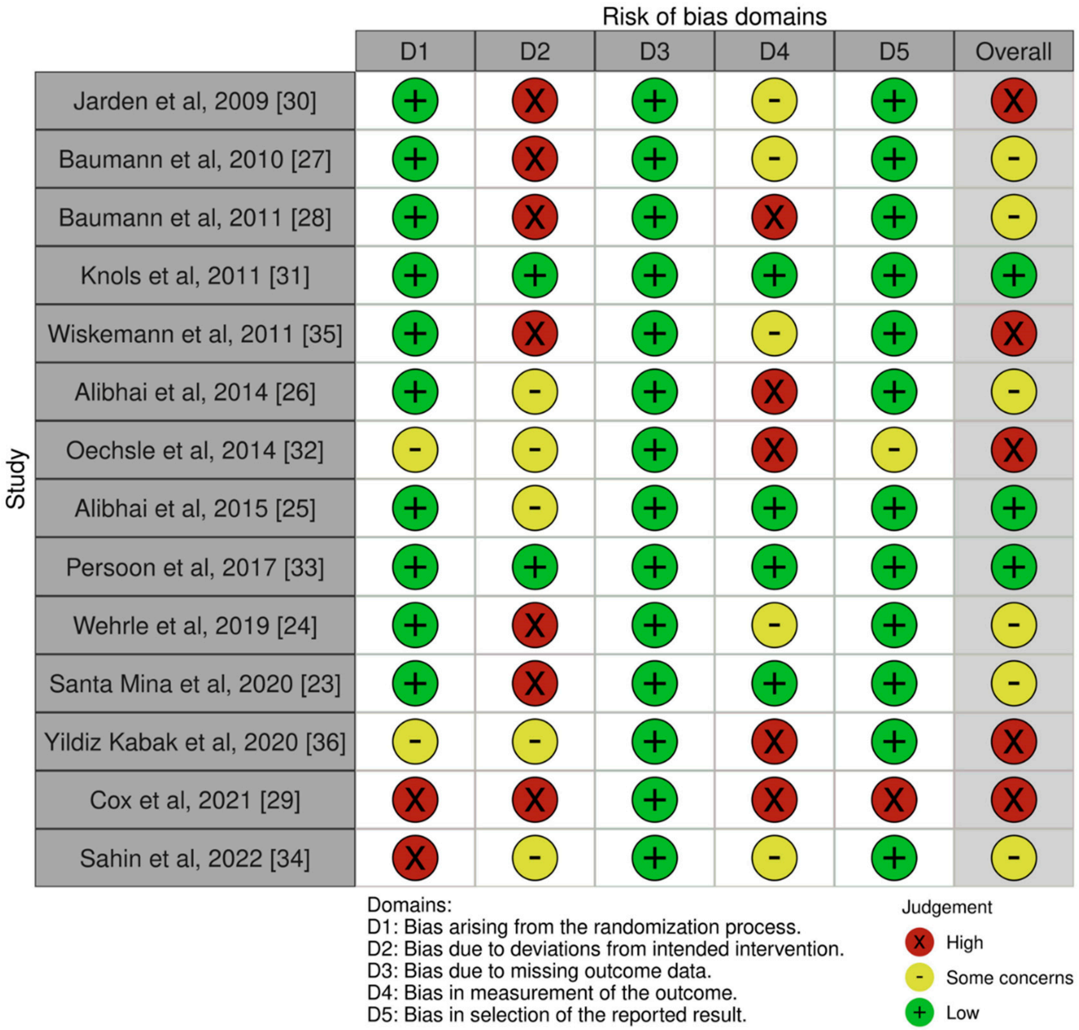

Table S3. Meta-analysis results on the effects of exercise on functioning domains and global health status of EORTC QLQ-C30

Effect size estimates for individual studies on functioning domains and global health status of EORTC-QLQC30												
Study		Exercise			Control			Weight	Weight (%)	Std. Mean difference; [95% CI]	Sig. (two tailed)	Std. Error
		Mean	SD	Sample size	Mean	SD	Sample Size					
Physical Functioning	Baumann et al, 2010 [27]	61.6	22.7	32	48.7	24.7	23	11.95	8.76	0.54 [0.00;1.08]	0.05	0.27
	Alibhai et al, 2015 [25]	75.3	30.7	57	76.8	29.6	24	15.01	11.01	-0.05[-0.52;0.42]	0.84	0.24
	Jarden et al, 2009 [30]	75.3	17.4	21	63.5	22.6	21	9.62	7.05	0.57 [-0.03;1.18]	0.06	0.31
	Wiskemann et al, 2011 [35]	73.7	19.6	52	63.5	22.2	53	21.21	15.55	0.48 [0.10;0.87]	0.01	0.20
	Baumann et al, 2011 [28]	65.9	16.5	24	59.6	22.9	23	10.89	7.98	0.31 [-0.25;0.88]	0.28	0.29
	Sahin et al, 2022 [34]	78.7	20.8	28	75.5	19.45	19	10.62	7.79	0.16 [-0.42;0.73]	0.60	0.29
	Persoon et al, 2017 [33]	83.1	19.1	54	84.1	15.3	55	22.36	16.40	-0.06 [-0.43;0.32]	0.76	0.19
	Knols et al, 2011 [31]	83.7	14.2	64	80.4	14	67	25.70	18.84	0.23 [-0.11;0.57]	0.18	0.17
	Alibhai et al, 2014 [26]	87.9	17.4	21	88.4	21.7	17	9.05	6.63	-0.03 [-0.65;0.60]	0.94	0.32
	General			353			302			0.23 [0.06;0.40]	0.001	0.09
	Heterogeneity (I^2) = 12.9%; Test for overall effect: Z=2.65											
Effect of exercise on Physical Functioning domain (PF)												
Study		Exercise			Control			Weight	Weight (%)	Std. Mean difference; [95% CI]	Sig. (two tailed)	Std. Error
		Mean	SD	Sample size	Mean	SD	Sample Size					
Role Functioning	Baumann et al, 2010 [27]	41.4	30.4	32	33.9	37.4	23	13.69	8.46	0.22[-0.31;0.75]	0.41	0.27

	Alibhai et al, 2015 [25]	54.1	48.6	57	46.5	44.12	24	17.17	10.61	0.16[-0.31;0.63]	0.51	0.24
	Jarden et al, 2009 [30]	52.0	35.3	21	39.2	42.5	21	10.77	6.45	0.32[-0.20;0.92]	0.29	0.30
	Wiskemann et al, 2011 [35]	45.0	28.5	52	43.8	32.8	53	26.63	16.45	0.04[-0.34;0.42]	0.84	0.19
	Baumann et al, 2011 [28]	45.1	24.8	24	37.5	40.1	23	12.07	7.46	0.23[-0.34;0.79]	0.43	0.29
	Sahin et al, 2022 [34]	82.2	20.7	28	87.2	20.2	19	11.63	7.10	-0.24[-0.01;0.34]	0.41	0.29
	Persoon et al, 2017 [33]	81.0	23.8	54	73.4	31.8	55	27.39	16.92	0.27[-0.11;0.64]	0.16	0.19
	Knols et al, 2011 [31]	68.7	24.2	64	61.3	26.0	67	32.77	20.26	0.29[-0.05;0.64]	0.09	0.17
	Alibhai et al, 2014 [26]	76.5	44.7	21	86.1	31.2	17	9.73	4.01	-0.24[-0.07;0.39]	0.46	0.32
	General			353			302			0.15[-0.00;0.31]	0.05	0.08
	Heterogeneity (I^2) = 0.0%; Test for overall effect: Z=1.94											
Effect of exercise on Role Functioning domain (RF)												
Study		Exercise			Control			Weight	Weight (%)	Std. Mean difference;	Sig. (two tailed)	Std. Error
		Mean	SD	Sample Size	Mean	SD	Sample Size			[95% CI]		
Emotional Functioning	Baumann et al, 2010 [27]	63.1	21.3	32	53	23.4	23	12.31	8.58	0.46[-0.09;1.00]	0.10	0.28
	Alibhai et al, 2015 [25]	79.7	29.2	57	78.8	34.1	24	15.66	10.92	0.03[-0.45;0.51]	0.90	0.24
	Jarden et al, 2009 [30]	79.4	19.8	21	66.7	23.0	21	9.61	6.70	0.59[-0.03;1.21]	0.06	0.32
	Wiskemann et al, 2011 [35]	70.0	24.2	52	60.5	25.1	53	23.02	16.05	0.39[-0.00;0.77]	0.05	0.20
	Baumann et al, 2011 [28]	70.3	18.6	24	57.8	22.9	23	10.68	7.45	0.60[-0.02;1.19]	0.04	0.30
	Sahin et al, 2022 [34]	75.4	27.6	28	84.1	26.8	19	10.63	7.41	-0.32[-0.90;0.27]	0.29	0.30
	Persoon et al, 2017 [33]	86.2	16.3	54	86	14.7	55	24.19	16.86	0.01[-0.36;0.39]	0.95	0.19
	Knols et al, 2011 [31]	76.1	24.9	64	72.6	22.2	67	28.35	19.76	0.15[-0.19;0.49]	0.40	0.18
	Alibhai et al, 2014 [26]	80.5	31.2	21	81.5	30.1	17	9.00	6.28	-0.03[-0.67;0.61]	0.92	0.33

	General			353			302			0.19[0.03;0.36]	0.02	0.08
	Heterogeneity (I^2) = 7.3%; Test for overall effect: Z= 2.32											
Effect of exercise on Emotional Functioning domain (EF)												
Study		Exercise			Control			Weight	Weight (%)	Std. Mean difference; [95% CI]	Sig. (two tailed)	Std. Error
		Mean	SD	Sample Size	Mean	SD	Sample Size					
Cognitive Functioning	Baumann et al, 2010 [27]	67.8	23.5	32	60.8	23.8	23	11.72	9.10	0.29[-0.24;0.82]	0.28	0.27
	Alibhai et al, 2015 [25]	83.7	29.6	57	72.9	27.95	24	14.12	10.96	0.37[-0.11;0.84]	0.13	0.24
	Jarden et al, 2009 [30]	79.4	25.4	21	61.8	24.1	21	9.16	7.11	0.70[0.09;1.31]	0.03	0.31
	Wiskemann et al, 2011 [35]	73.8	22.3	52	71.3	19.6	53	20.19	15.68	0.12[-0.26;0.50]	0.54	0.19
	Baumann et al, 2011 [28]	71.6	24.1	24	68.8	22.9	23	10.60	8.23	0.12[-0.45;0.68]	0.68	0.29
	Sahin et al, 2022 [34]	77.1	24.5	28	87.5	23.8	19	10.09	7.83	-0.42[-1.00;0.16]	0.15	0.30
	Persoon et al, 2017 [33]	83.7	18.0	54	82.3	20.4	55	20.78	16.13	0.07[-0.30;0.45]	0.70	0.19
	Knols et al, 2011 [31]	87.9	16.7	64	79.2	21.7	67	23.36	18.14	0.43[0.09;0.77]	0.01	0.18
	Alibhai et al, 2014 [26]	82.0	36.7	21	84.6	24.8	17	8.77	6.81	-0.08[-0.71;0.55]	0.80	0.32
	General			353			302			0.20[0.02;0.37]	0.03	0.09
	Heterogeneity (I^2) = 17.2%; Test for overall effect: Z= 2.22											
Effect of exercise on Cognitive Functioning domain (CF)												
Study		Exercise			Control			Weight	Weight (%)	Std. Mean difference; [95% CI]	Sig. (two tailed)	Std. Error
		Mean	SD	Sample Size	Mean	SD	Sample Size					
Social Functioning	Baumann et al, 2010 [27]	47.7	35.6	32	40.3	39.6	23	13.69	8.47	0.20[-0.33;0.72]	0.47	0.27
	Alibhai et al, 2015 [25]	52.9	47.6	57	48.5	46.17	24	17.17	10.63	0.09[-0.38;0.56]	0.70	0.24
	Jarden et al, 2009 [30]	71.6	30.5	21	54.9	23.4	21	10.42	6.45	0.60[-0.00;1.21]	0.05	0.31
	Wiskemann et al, 2011 [35]	49.2	32.5	52	46.7	29.3	53	26.54	16.42	0.08[-0.30;0.46]	0.68	0.19

	Study	Exercise Mean	Exercise SD	Exercise Sample Size	Control Mean	Control SD	Control Sample Size	Weight	Weight (%)	Std. Mean difference; [95% CI]	Sig. (two tailed)	Std. Error
	Baumann et al, 2011 [28]	52.0	30.0	24	39.6	42.6	23	11.97	7.41	0.33[-0.23;0.90]	0.25	0.29
	Sahin et al, 2022 [34]	77.6	22.0	28	85.9	21.36	19	11.50	7.12	-0.38[-0.95;0.20]	0.20	0.29
	Persoon et al, 2017 [33]	86.0	20.3	54	83.7	20.4	55	27.51	17.03	0.11[-0.26;0.49]	0.56	0.19
	Knols et al, 2011 [31]	71.3	28.6	64	72.9	24.9	67	32.99	20.42	-0.06[-0.40;0.28]	0.73	0.17
	Alibhai et al, 2014 [26]	44.1	18.3	21	43.7	21.51	17	9.79	6.06	0.02[-0.61;0.65]	0.95	0.32
	General			353			302			0.08[-0.07;0.24]	0.28	0.08
	Heterogeneity (I^2) = 0.2%; Test for overall effect: Z= 1.07											

Effect of exercise on Social Functioning domain (SF)

Study		Exercise			Control			Weight	Weight (%)	Std. Mean difference; [95% CI]	Sig. (two tailed)	Std. Error
		Mean	SD	Sample Size	Mean	SD	Sample Size					
Global Health Status	Baumann et al, 2010 [27]	57.5	21.4	32	45.2	23.3	23	8.26	9.86	0.55[0.01-1.08]	0.05	0.27
	Alibhai et al, 2015 [25]	63.2	39.2	57	52.4	40.9	24	9.59	11.43	0.27[-0.20;0.74]	0.27	0.24
	Jarden et al, 2009 [30]	61.3	17.2	21	52.9	16.7	21	7.13	8.51	0.49[-0.12;1.09]	0.11	0.31
	Wiskemann et al, 2011 [35]	61.7	22.2	52	57.1	17.3	53	11.96	14.27	0.23[-0.15;0.61]	0.24	0.19
	Baumann et al, 2011 [28]	68.6	11.2	24	56.3	17.6	23	7.40	8.83	0.82[0.24;1.41]	0.01	0.30
	Sahin et al, 2022 [34]	61.8	19.3	28	69.8	18.7	19	7.52	8.97	-0.41[-0.99;0.17]	0.16	0.30
	Persoon et al, 2017 [33]	75	18.7	54	73.4	18.4	55	12.19	14.55	0.09[-0.29;0.46]	0.65	0.19
	Knols et al, 2011 [31]	73.7	14.9	64	67.7	15.7	67	13.06	15.58	0.39[0.05;0.73]	0.03	0.18
	Alibhai et al, 2014 [26]	71.1	20.9	21	78.9	26.65	17	6.71	8.00	-0.32[-0.95;0.31]	0.31	0.32
	General			353			302			0.24[0.03;0.46]	0.03	0.11
	Heterogeneity (I^2) = 2.21%; Test for overall effect: Z= 44.2											

Effect of exercise on Global Health Status domain (GHS)

Table S4. Meta-analysis results on the effects of exercise on symptoms domains of EORTC QLQ-C30

Effect size estimates for individual studies on symptoms domains of EORTC-QLQC30												
Study		Exercise			Control			Weight	Weight (%)	Std. Mean difference [95% CI]	Sign. Two tailed	Std Error
		Mean	SD	Sample Size	Mean	SD	Sample Size					
Fatigue	Baumann et al, 2010 [27]	51.0	23.3	32	61.3	26.6	23	13.49	12.64	-0.41 [-0.94;0.12]	0.13	0.27
	Jarden et al, 2009 [30]	50.3	24.6	21	58.8	260	21	10.76	10.08	-0.33[-0.93;0.27]	0.28	0.30
	Wiskemann et al, 2011 [35]	49.7	28.9	52	60.8	29.2	53	26.16	24.50	-0.38 [-0.76;0.00]	0.08	0.20
	Baumann et al, 2011 [28]	43.8	22.7	24	52.8	27.1	23	11.96	11.20	-0.35 [-0.92;0.21]	0.22	0.29
	Sahin et al, 2022 [34]	66.5	22.0	28	68.8	21.4	19	11.69	10.95	-0.10[-0.68;0.47]	0.72	0.29
	Knols et al, 2011 [31]	31.6	22.1	64	38.3	20.0	67	32.71	30.63	-0.32[-0.66;0.03]	0.07	0.17
	General			221			206			-0.33[-0.52;-0.14]	0.001	0.10
	Heterogeneity (I^2) = 0.0%; Test for overall effect: Z=-3.37											
Effect of exercise on Fatigue												
Study		Exercise			Control			Weight	Weight (%)	Std. Mean difference [95% CI]	Sign. Two tailed	Std Error
		Mean	SD	Sample Size	Mean	SD	Sample Size					
Nausea/Vomiting	Baumann et al, 2010 [27]	26.4	33.5	32	30.6	29.5	23	13.74	12.77	-0.13[-0.66;0.40]	0.63	0.27
	Jarden et al, 2009 [30]	14.7	16.5	21	24.5	31.3	21	10.71	9.96	-0.38[-0.98;0.21]	0.21	0.31
	Wiskemann et al, 2011 [34]	13.8	22.3	52	19.2	26.6	53	26.48	24.61	-0.22[-0.60;0.16]	0.26	0.19
	Baumann et al, 2011 [28]	10.8	18.6	24	13.5	17.7	23	12.12	11.26	-0.15[-0.71;0.42]	0.61	0.29
	Sahin et al, 2022 [34]	81.7	22	28	86.5	21.3	19	11.64	10.82	-0.22[-0.79;0.36]	0.46	0.29
	Knols et al, 2011 [31]	3.7	9.9	64	6.5	13.4	67	32.89	30.57	-0.24[-0.58;0.11]	0.18	0.17
	General			221			206			-0.22[-0.41;-0.03]	0.02	0.10
	Heterogeneity (I^2) = 0.0%; Test for overall effect: Z=-2.28											
Effect of exercise on Nausea/Vomiting												

Study		Exercise			Control			Weight	Weight (%)	Std. Mean difference [95% CI]	Sign. Two tailed	Std Error
		Mean	SD	Sample Size	Mean	SD	Sample Size					
Pain	Baumann et al, 2010 [27]	33.3	29.2	32	40.3	28.8	23	13.68	12.81	-0.24[-0.77;0.29]	0.38	0.27
	Jarden et al, 2009 [30]	23.5	27	21	36.3	28.4	21	10.64	9.96	-0.45[-1.05;0.15]	0.14	0.31
	Wiskemann et al, 2011 [35]	25	31.8	52	35.8	28.9	53	26.23	24.57	-0.35[-0.74;0.03]	0.07	0.20
	Baumann et al, 2011 [28]	22.5	25.6	24	32.3	26.2	23	11.94	11.19	-0.37[-0.94;0.20]	0.20	0.29
	Sahin et al, 2022 [34]	74.5	25.5	28	82.7	24.6	19	11.57	10.84	-0.32[-0.90;0.26]	0.28	0.29
	Knols et al, 2011 [31]	17.8	27.1	64	26.8	28.4	67	32.69	30.63	-0.32[-0.66;0.02]	0.07	0.17
	General			221			206			-0.34[-0.53;-0.15]	0.00	0.10
	Heterogeneity (I^2) = 0.0%; Test for overall effect: Z=-3.48											

Effect of exercise on Pain

Study		Exercise			Control			Weight	Weight (%)	Std. Mean difference [95% CI]	Sign. Two tailed	Std Error
		Mean	SD	Sample Size	Mean	SD	Sample Size					
Dyspnoea	Baumann et al, 2010 [27]	29.9	28.7	32	39.8	33.8	23	13.61	12.65	-0.32[-0.85;0.22]	0.24	0.27
	Jarden et al, 2009 [30]	23.5	28.3	21	33.3	37.3	21	10.79	10.03	-0.29[-0.89;0.31]	0.34	0.30
	Wiskemann et al, 2011 [35]	30.0	25.9	52	33.3	31.3	53	26.59	24.72	-0.11[-0.49;0.27]	0.56	0.19
	Baumann et al, 2011 [28]	27.4	24.3	24	29.2	29.5	23	12.14	11.29	-0.07[-0.63;0.50]	0.82	0.29
	Sahin et al, 2022 [34]	80.1	29.9	28	78.9	29.0	19	11.71	10.88	0.04[-0.53;0.61]	0.89	0.29
	Knols et al, 2011 [31]	21.8	24.6	64	29.2	23.0	67	32.73	30.42	-0.31[-0.65;0.03]	0.08	0.17
	General			221			206			-0.19[-0.38;-0.01]	0.04	0.10
	Heterogeneity (I^2) = 0.0%; Test for overall effect: Z=-2.01											

Effect of exercise on Dyspnoea

Study		Exercise			Control			Weight	Weight (%)	Std. Mean difference [95% CI]	Sign. Two tailed	Std Error
		Mean	SD	Sample Size	Mean	SD	Sample Size					

Insomnia	Baumann et al, 2010 [27]	46.0	36.1	32	39.8	31.5	23	13.68	12.83	0.18[-0.35-0.71]	0.51	0.27
	Jarden et al, 2009 [30]	21.6	26.2	21	41.2	32.3	21	10.33	9.96	-0.65[-1.26;-0.04]	0.04	0.31
	Wiskemann et al, 2011 [35]	30.8	32.4	52	35.8	31.5	53	26.42	24.77	-0.16[-0.54;0.22]	0.42	0.19
	Baumann et al, 2011 [28]	35.3	32.2	24	41.7	35.5	23	12.07	11.32	-0.19[-0.75;0.38]	0.52	0.29
	Sahin et al, 2022 [34]	72.2	27.6	28	80.1	26.8	19	11.57	10.85	-0.28[-0.86;0.29]	0.33	0.29
	Knols et al, 2011 [31]	23.0	27.4	64	31.5	30.8	67	32.57	30.54	-0.29[-0.63;0.05]	0.10	0.17
	General			221			206			-0.22[-0.41;-0.03]	0.02	0.10
	Heterogeneity (I^2) = 0.3%; Test for overall effect: Z=-2.26											
Effect of exercise on Insomnia												
Study		Exercise			Control			Weight	Weight (%)	Std. Mean difference [95% CI]	Sign. Two tailed	Std Error
		Mean	SD	Sample Size	Mean	SD	Sample Size					
Appetite Loss	Baumann et al, 2010 [27]	48.3	35.2	32	45.2	33.9	23	13.73	12.83	0.09[-0.44;0.62]	0.74	0.27
	Jarden et al, 2009 [30]	39.2	33.8	21	60.8	33.8	21	10.38	9.70	-0.63[-1.23;-0.02]	0.04	0.31
	Wiskemann et al, 2011 [35]	31.7	35.4	52	42.5	37.7	53	26.26	24.53	-0.29[-0.67;0.09]	0.13	0.19
	Baumann et al, 2011 [28]	31.4	24.9	24	27.1	27.8	23	12.09	11.29	0.16[-0.40;0.72]	0.58	0.29
	Sahin et al, 2022 [34]	79.6	22.6	28	81.4	22.0	19	11.68	10.91	-0.08[0.65;0.49]	0.79	0.29
	Knols et al, 2011 [31]	8.6	17.2	64	10.7	22.1	67	32.93	30.75	-0.11[-0.45;0.24]	0.55	0.17
	General			221			206			-0.14[-0.33;0.05]	0.14	0.10
	Heterogeneity (I^2) = 0.2%; Test for overall effect: Z=-1.49											
Effect of exercise on Appetite Loss												
Study		Exercise			Control			Weight	Weight (%)	Std. Mean difference [95% CI]	Sign. Two tailed	Std Error
		Mean	SD	Sample Size	Mean	SD	Sample Size					
Constipation	Baumann et al, 2010 [31]	5.9	13.0	32	7.5	20.6	23	13.37	14.26	-0.10[-0.63;0.44]	0.73	0.27

	Jarden et al, 2009 [30]	3.9	11.1	21	13.7	29.0	21	10.23	10.91	-0.14[-1.05;0.17]	0.16	0.31
	Wiskemann et al, 2011 [35]	4.2	17.2	52	5.8	14.9	53	26.21	27.97	-0.10[-0.48;0.28]	0.61	0.20
	Sahin et al, 2022 [34]	80.1	29.4	28	79.5	28.4	19	11.32	12.08	0.02[-0.56;0.60]	0.95	0.30
	Knols et al, 2011 [31]	4.6	17.0	64	7.7	16.8	67	32.59	34.78	-0.18[-0.53;0.16]	0.30	0.18
	General			197			183			-0.15[-0.35;0.05]	0.15	0.10
	Heterogeneity (I^2) =0.0%; Test for overall effect: Z=-1.45											
Effect of exercise on Constipation												
Study		Exercise			Control			Weight	Weight (%)	Std. Mean difference [95% CI]	Sign. Two tailed	Std Error
		Mean	SD	Sample Size	Mean	SD	Sample Size					
Diarrhea	Baumann et al, 2010 [27]	34.5	39.3	32	41.9	36.5	23	13.31	12.80	-0.19[-0.73;0.35]	0.49	0.27
	Jarden et al, 2009 [30]	19.6	29.0	21	41.2	30.1	21	9.80	9.43	-0.72[-1.34;-0.09]	0.02	0.32
	Wiskemann et al, 2011 [35]	10.8	25.5	52	18.3	27.2	53	25.26	24.97	-0.28[-0.67;0.10]	0.15	0.20
	Baumann et al, 2011 [28]	23.5	28.3	24	20.8	26.9	23	11.73	11.28	0.10[-0.48;0.67]	0.74	0.29
	Sahin et al, 2022 [34]	88.9	21.8	28	88.5	21.2	19	11.31	10.88	0.02[-0.56;0.60]	0.95	0.30
	Knols et al, 2011 [31]	8.6	18.3	64	18.5	24.6	67	31.86	30.64	-0.45[-0.80;-0.11]	0.01	0.18
	General			221			206			-0.29[-0.48;-0.10]	0.00	0.10
	Heterogeneity (I^2) =0.0%; Test for overall effect: Z=-2.94											
Effect of exercise on Diarrhea												
Study		Exercise			Control			Weight	Weight (%)	Std. Mean difference [95% CI]	Sign. Two tailed	Std Error
		Mean	SD	Sample Size	Mean	SD	Sample Size					
Financial Difficulties	Baumann et al, 2010 [27]	23.8	32.5	32	40.9	35.2	23	13.36	17.19	-0.50[-1.04;0.04]	0.07	0.27
	Baumann et al, 2011 [28]	27.5	33.8	24	41.7	37.5	23	11.92	15.33	-0.39[-0.96;0.18]	0.18	0.29
	Sahin et al, 2022 [34]	76.9	30.3	28	77.6	29.4	19	11.71	15.06	-0.02[-0.60;0.55]	0.94	0.29

	Knols et al, 2011 [31]	14.4	27.3	64	18.5	27.6	67	33.03	42.48	-0.15[-0.49;0.19]	0.39	0.17
	General			148			132			-0.25[-0.48;-0.03]	0.03	0.11
	Heterogeneity (I^2) =0.0%; Test for overall effect: Z=-2.23											
	Effect of exercise on Financial Difficulties											
